# The prognostic value and model construction of inflammatory markers for patients with non-small cell lung cancer

**DOI:** 10.1038/s41598-024-57814-4

**Published:** 2024-03-30

**Authors:** Wanting Xu, Xinya Liu, Ci Yan, Gulinurayi Abdurahmane, Jiayina Lazibiek, Yan Zhang, Mingqin Cao

**Affiliations:** 1 Department of Epidemiology and Health Statistics, School of Public Health, Xinjiang Medical University, Urumqi, 830000 People’s Republic of China; 2 The Affiliated Tumor Hospital, Xinjiang Medical University, Urumqi, 830000 People’s Republic of China

**Keywords:** Cancer, Biomarkers, Oncology

## Abstract

The aim of this study was to investigate and analyse the predictive value of systemic inflammatory markers based on peripheral blood biomarkers for the prognosis of non-small cell lung cancer (NSCLC) patients. Based on a retrospective monitoring cohort of 973 NSCLC patients from an Affiliated Tumor Hospital from 2012 to 2023. The log-rank test and Cox proportional risk regression model were used to identify independent prognostic inflammatory markers. Subsequently, a nomogram prediction model was constructed and evaluated. The results of multivariate Cox regression analysis showed that patients with high NLR group (*HR* = 1.238, 95% CI 1.015–1.510, *P* = 0.035), and high CAR group (*HR* = 1.729, 95% CI 1.408–2.124, *P* < 0.001) were risk factors affecting the prognosis of NSCLC patients. The nomogram that includes age, tumor stage, smoking history, BMI, NLR, and CAR can effectively predict the prognosis of NSCLC patients.The inflammatory markers NLR and CAR, which combine inflammatory and nutritional status, are effective predictors of the prognosis of NSCLC patients. The combination of clinical information and these easily accessible inflammatory markers has significant research value for prognostic assessment, clinical treatment, and follow-up monitoring of NSCLC patients.

Lung cancer is the leading cause of cancer-related deaths worldwide. Global cancer statistics from 2020 reveal that there were 2.207 million new cases of lung cancer, making it the second most common cancer after breast cancer. Additionally, there were 1.796 million deaths from lung cancer, making it the deadliest cancer type^1,2^. Non-small cell lung cancer (NSCLC) is the most common subtype of lung cancer, accounting for about 80–85% of all cases. The majority of NSCLC cases are diagnosed at an advanced stage, either locally advanced or with distant metastases, resulting in a poor prognosis^3^. The 5-year survival rate for patients with stage I NSCLC is 68.4%, while the 5-year survival rate for patients with stage IV NSCLC is less than 10%^4^. Currently, the methods for diagnosing and monitoring the progression of lung cancer often involve a combination of clinical manifestations, imaging studies, and biopsies performed on the affected areas. However, pathological biopsies can be traumatic for patients and result in long diagnostic cycles. Therefore, developing a prognostic prediction model for lung cancer using simple, convenient, cost-effective, and reliable peripheral blood biomarkers holds significant clinical value and promising application prospects.

Based on current research, there is evidence to suggest that systemic inflammation is associated with tumor characteristics such as proliferation, invasion and metastasis. Inflammation plays a significant role in tumor formation and growth^5^. Systemic inflammatory markers, such as neutrophils, platelets, lymphocytes, monocytes, C-reactive protein and albumin, can effectively reflect the systemic inflammatory status of tumors. They also provide important information for prognostic prediction of NSCLC and other tumors^6,7^. This study used a 10-year follow-up cohort of NSCLC patients from a specialist tumor hospital, along with information and data from the hospital information system. Screening prognostic information, clinical characteristics, and inflammation-related biomarkers of patients to explore and analyze the predictive value of inflammatory markers for the prognosis of NSCLC patients. This study aims to provide a reference basis for early detection of poor prognostic outcomes and the timely implementation of precise treatment and intervention.

## Materials and methods

### Study population

The study focused on 973 NSCLC patients who were diagnosed and hospitalized at the Affiliated Tumor Hospital of Xinjiang Medical University from 2012 to 2023. All methods were carried out in accordance with the Declaration of Helsinki (as revised in 2013). All experimental protocols were approved by the local ethics committee of Affiliated Tumor Hospital of Xinjiang Medical University. Informed consent was obtained from all subjects and/or their legal guardian(s).

The tumor staging of the NSCLC patients was based on the seventh edition of the AJCC staging criteria. The inclusion criteria for the study subjects were as follows: (1) age ≥ 18 years; (2) diagnosed with NSCLC through cytology or pathology; (3) received first-line treatment, including surgery, chemotherapy, or targeted therapy; (4) had complete clinical data and were willing to participate in follow-up. The exclusion criteria were as follows: (1) non-compliance or refusal to follow-up or missing clinical data; (2) concurrent presence of other malignant tumors; (3) concurrent active tuberculosis or other severe infectious diseases; (4) concurrent severe diseases of the heart, lungs, liver, and hematopoietic system; (5) severe brain disease or mental illness; (6) history of organ transplantation.

### Data collection

To retrospectively collect clinicopathological features of NSCLC patients, including age, gender, tumor stage, primary site, type of pathology, education level, occupation, smoking history, drinking history, height, weight, Body Mass Index (BMI), Body Surface Area (BSA), Karnofsky Performance Scores (KPS), and Eastern Cooperative Oncology Group Performance Status (ECOG-PS).

The ECOG scale was used to evaluate each patient's performance status(PS) at enrollment. The ECOG-PS were transformed from KPS data according to the criteria as follows^8^: KPS score = 100 (ECOG-PS 0), KPS score = 80–90 (ECOG-PS 1), KPS score = 60–70 (ECOG-PS 2), KPS score = 40–50 (ECOG-PS 3), KPS score = 0–30 (ECOG-PS 4). ECOG 0–1 was classified as the good PS (ECOG 0 or 1), and ECOG 2–4 was defined as poor PS (ECOG ≥ 2).

Hematological indexes at the time of first diagnosis included neutrophils (N), monocytes (M), lymphocytes (L), platelets (P), serum albumin (ALB), and C-reactive protein (CRP). Using hematological indicators to calculate inflammatory markers, eight systemic inflammatory markers were assessed in this study, as shown in Table 1.

**Table 1:** Eight inflammatory markers evaluated in this study.

Inflammatory markers	Inflammatory markers formulas
Neutrophil-to-lymphocyte ratio (NLR)	Neutrophil/lymphocyte
Platelet-to-lymphocyte ratio (PLR)	Platelet/lymphocyte
Lymphocyte-to-monocyte ratio (LMR)	Lymphocyte/monocyte
Systemic immune inflammation index (SII)	Platelet × neutrophil/lymphocyte
Prognostic nutrition index (PNI)	Lymphocyte × 5 + Albumin
Systemic inflammatory response index (SIRI)	Neutrophil × monocyte/lymphocyte
C-reactive protein-to-Albumin ratio (CAR)	C-reactive protein / Albumin
Modified Glasgow Prognostic Score (mGPS)	C-reactive protein ≤ 10 mg/L: 0 score
	C-reactive protein > 10 mg/L and Albumin ≥ 35 g/L: 1 score
	C-reactive protein > 10 mg/L and Albumin < 35 g/L: 2 score

### Follow up

The survival and disease progression of the NSCLC patients after discharge were monitored using the electronic diagnosis and treatment medical record management system at the Affiliated Tumor Hospital of Xinjiang Medical University. Follow-up was conducted through telephone, outpatient clinic visits, and WeChat communication. Investigate the patient's condition or survival and provide a summary. The follow-up endpoint of this study was mortality, and overall survival (OS) was defined as the duration from the beginning of initial admission to either death or the last follow-up. The follow-up period extended until February 25, 2023, during which a total of 973 complete cases were monitored.

### Statistical analysis

Data were analyzed and plotted using SPSS 26.0, R language software (version 4.2.3), and RStudio software. The life-table method was used to calculate survival rates and plot survival curves for NSCLC patients. The Receiver operating characteristic (ROC) curves were used to determine the optimal group cut-off values for the eight inflammatory markers and assess their predictive value for the prognosis of NSCLC patients. Survival curves for the eight inflammatory markers were plotted using Kaplan–Meier survival analysis. Log-Rank tests were conducted to analyze the survival differences between the different groups. Univariate and multivariate Cox proportional hazards regression model analyses were conducted to investigate the factors influencing the prognosis of NSCLC. The chi-square test (*χ*^2^) and the Mann–Whitney U test were used to analyze the relationship between inflammatory markers and NSCLC disease progression. The patient survival prediction model nomogram was constructed using R 4.2.3 software and RStudio software. The consistency index (C-index) was calculated using the Bootstrap method with 1000 repeated samples to evaluate the prediction ability of the nomogram. Additionally, the calibration curve was used to assess the degree of agreement between the actual and predicted results of the model. Differences were considered statistically significant at *P* < 0.05 (two-tailed).

## Results

### Description of clinicopathological features

A total of 973 NSCLC patients were included in this study. Among them, 57 (5.86%) were aged ≤ 45 years, 458 (47.07%) were aged 46–64 years, and 458 (47.07%) were aged ≥ 65 years. The average age of the patients was 63 years, with a range from 26 to 92 years. There were 625 males (64.23%) and 348 females (35.77%). The tumor stage was classified as stage I in 140 cases (14.39%), stage II in 56 cases (5.76%), stage III in 218 cases (22.40%) and stage IV in 559 cases (57.45%). There were 5 cases of large cell carcinoma (0.51%), 681 cases of adenocarcinoma (69.99%), and 287 cases of squamous carcinoma (29.50%). There were 532 cases (54.68%) with a history of smoking and 311 cases (31.96%) with a history of drinking. The mean BMI was 23.97 (ranging from 13.9 to 41.0). 929 (95.48%) with ECOG-PS 0–1, while 44 (4.52%) with ECOG-PS ≥ 2.

A total of eight inflammatory markers, NLR, PLR, LMR, SII, PNI, SIRI, CAR, and mGPS, were included in this study. Among these markers, NLR, PLR, LMR, SII, PNI, SIRI, and CAR were expressed as quantitative data, with median values of 3.07, 172.32, 3.03, 788.80, 52.45, 1.54, and 0.20 × 10^9/L, respectively. The mGPS showed qualitative grouping as follows: 501 patients (51.49%) had an mGPS of 0 score, 350 patients (35.97%) had an mGPS of 1 score, and 122 patients (12.54%) had an mGPS of 2 score. Other details are shown in Table 2.

**Table 2:** The clinicopathological features in NSCLC patients.

Characteristic	Levels	Overall *n* = 973
Age* n *(%)	≤ 45	57 (5.86)
	46– 64	458 (47.07)
	≥ 65	458 (47.07)
Gender *n *(%)	Female	348 (35.77)
	Male	625 (64.23)
Stage *n *(%)	I	140 (14.39)
	II	56 (5.76)
	III	218 (22.40)
	IV	559 (57.45)
Primary site *n *(%)	Hilum	66 (6.78)
	Bronchial	38 (3.91)
	Upper lobe of the lungs	485 (49.85)
	Middle lobe of the lungs	63 (6.47)
	Lower lobe of the lungs	321 (32.99)
Pathology *n *(%)	Large cell carcinoma	5 (0.51)
	Adenocarcinoma	681 (69.99)
	Squamous Carcinoma	287 (29.50)
Education *n *(%)	Elementary and below	305 (31.35)
	Middle School	303 (31.14)
	High School	169 (17.37)
	College and above	196 (20.14)
Occupation *n *(%)	Self-employed	38 (3.91)
	Workers	119 (12.23)
	Farmers and herdsmen	178 (18.29)
	Others	199 (20.45)
	Institutionalized	67 (6.89)
	Retired	254 (26.10)
	Unemployed	53 (5.45)
	Employee	65 (6.68)
Smoking history *n *(%)	No	441 (45.32)
	Yes	532 (54.68)
Drinking history *n *(%)	No	662 (68.04)
	Yes	311 (31.96)
BMI	(median (IQR))	23.67 (21.72, 25.95)
BSA	(median (IQR))	1.71 (1.60, 1.83)
KPS score	(median (IQR))	90 (90, 90)
ECOG-PS	0–1	929 (95.48)
	≥ 2	44 (4.52)
Neutrophil	(median (IQR))	4.71 (3.50, 6.24)
Lymphocyte	(median (IQR))	1.49 (1.14, 1.90)
Monocyte	(median (IQR))	0.52 (0.39, 0.68)
Platelet	(median (IQR))	254.00 (206.00, 317.00)
CRP	(median (IQR))	9.36 (2.73, 38.77)
ALB	(median (IQR))	43.80 (38.60, 54.45)
NLR	(median (IQR))	3.07 (2.08, 4.64)
PLR	(median (IQR))	172.32 (127.37, 232.71)
LMR	(median (IQR))	3.03 (1.98, 4.18)
SII	(median (IQR))	788.80 (489.04, 1314.71)
PNI	(median (IQR))	52.45 (46.10, 62.50)
SIRI	(median (IQR))	1.54 (0.88, 2.86)
CAR	(median (IQR))	0.20 (0.60, 0.94)
mGPS* n *(%)	0 score	501 (51.49)
	1 score	350 (35.97)
	2 score	122 (12.54)

### Survival status of NSCLC patients

**Figure 1:**
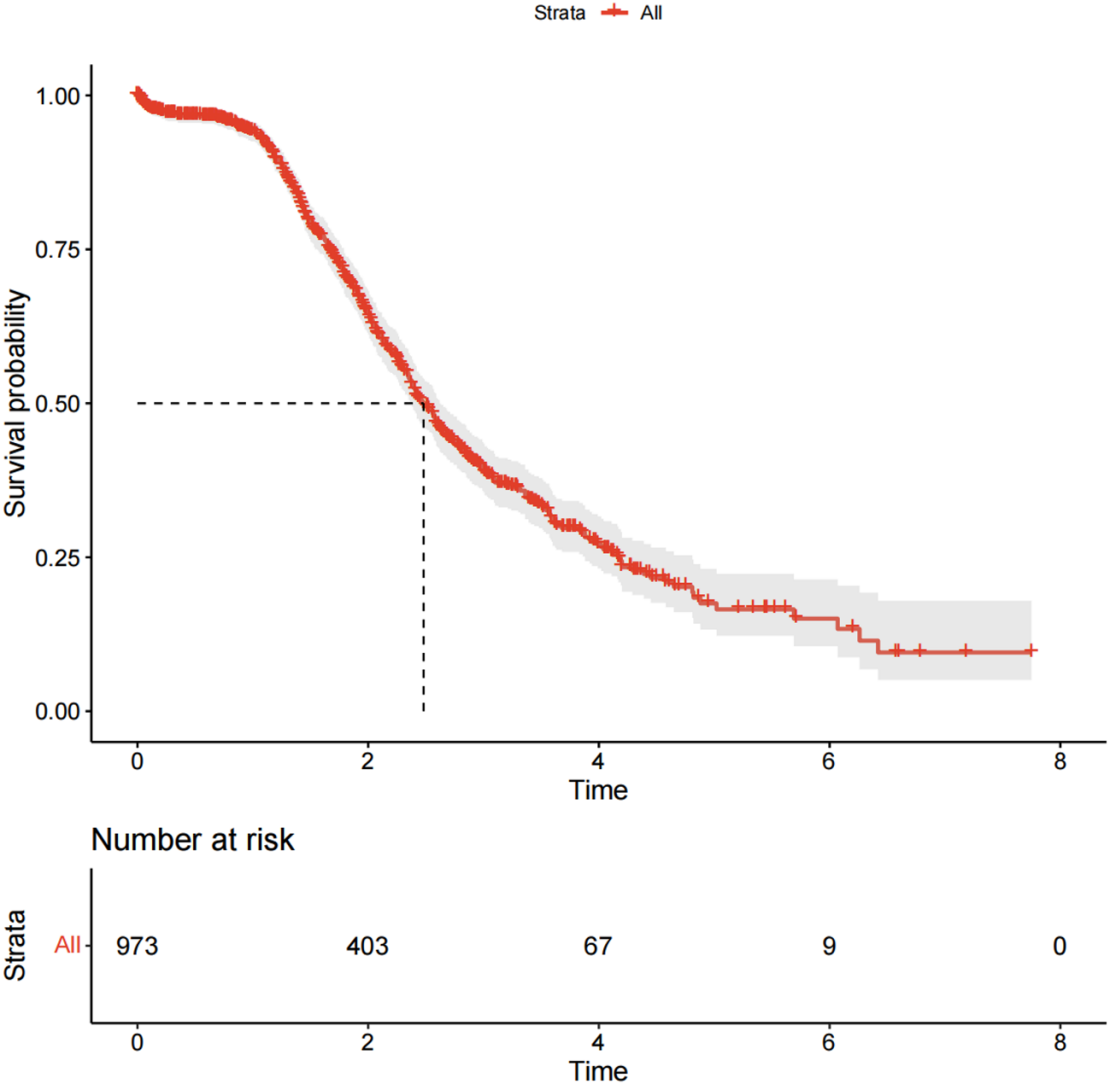
OS curve of NSCLC patients.

The follow-up cut-off date for this study cohort was February 25, 2023. By the end of the follow-up, a total of 460 patients died from NSCLC, accounting for 47.28% of the total number of NSCLC patients. Additionally, 513 patients with NSCLC, accounting for 52.72% of the total number of patients with NSCLC, either survived or were lost to follow-up. The overall median survival time for all patients included in this study was 1.75 (0.99, 2.64) years. The 1-year survival rate was 94.07%, the 3-year survival rate was 39.60%, and the 5-year survival rate was 18.94%. Specific survival is detailed in Table 3 and Fig. 1 for the survival curve.

**Table 3:** Survival of NSCLC patients at different time points and their corresponding standard errors.

No	Follow-up year	Number of terminal events	Number withdrawing during interval	Number entering interval	Number exposed to risk	Proportion terminating	Proportion surviving	Cumulative proportion surviving at end of interval	Std. error of cumulative surviving at end of interval
1	0 ~	52	192	973	877.0	0.06	0.94	0.94	0.01
2	1 ~	205	122	729	668.0	0.31	0.69	0.65	0.02
3	2 ~	140	91	402	356.5	0.39	0.61	0.40	0.02
4	3 ~	42	62	171	140.0	0.30	0.70	0.28	0.02
5	4 ~	16	33	67	50.5	0.32	0.68	0.19	0.02
6	5 ~	2	7	18	14.5	0.14	0.86	0.16	0.03
7	6 ~	3	4	9	7.0	0.43	0.57	0.09	0.03
8	7 ~	0	2	2	1.0	0.00	1.00	0.09	0.03

### Cut-off value analysis of inflammatory markers and their prognostic predictive value in NSCLC patients

**Figure 2:**
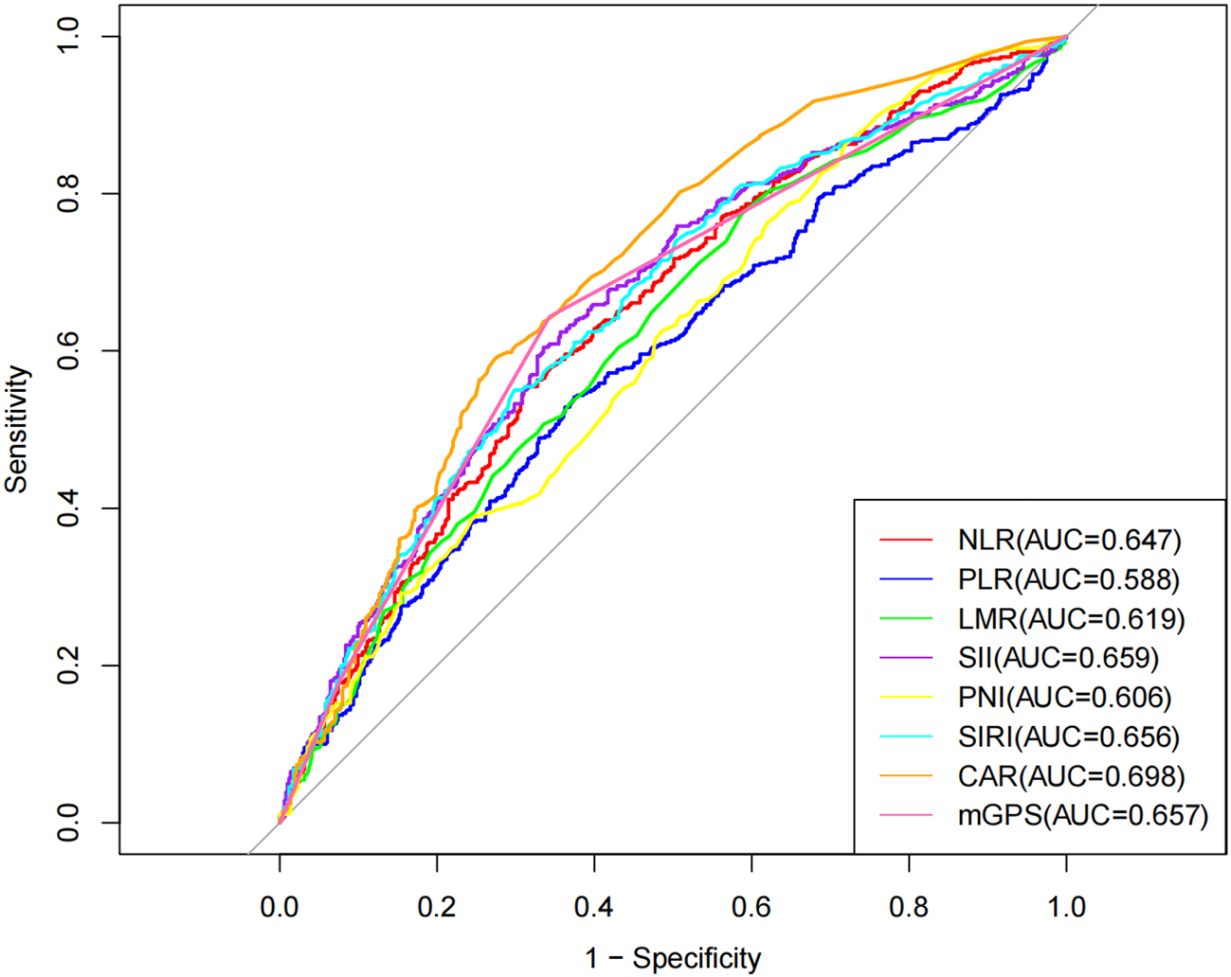
ROC curves for inflammatory markers in predicting prognosis among NSCLC patients.

The optimal cut-off values for NLR, PLR, LMR, SII, PNI, SIRI, and CAR were calculated using the Jordan's index and were determined to be 3.42, 179.16, 3.85, 829.96, 64.08, 1.88 and 0.32, respectively. Based on the optimal cut-off value, the patients were divided into two groups: the low inflammatory markers group (inflammatory markers ≤ optimal cut-off value) and the high inflammatory markers group (inflammatory markers > optimal cut-off value). The ROC curves were plotted for NLR, PLR, LMR, SII, PNI, SIRI, CAR, and mGPS to predict the prognosis of NSCLC patients. The AUC values were 0.647, 0.588, 0.619, 0.659, 0.606, 0.656, 0.698, and 0.657, respectively. All AUC values were greater than 0.5, indicating predictive ability. Among them, CAR had the highest AUC value and performed the best in predicting patient prognosis. See Table 4 and Fig. 2.

**Table 4:** Prognostic and predictive value of inflammatory markers in NSCLC patients.

Inflammatory markers	AUC	95% CI	Sensitivity (%)	Specificity (%)	Youden index	Optimal cut-off value
NLR	0.647	(0.612, 0.681)	55.2	68.8	0.240	3.42
PLR	0.588	(0.552, 0.624)	54.1	62.6	0.167	179.16
LMR	0.619	(0.584, 0.654)	39.8	79.6	0.194	3.85
SII	0.659	(0.625, 0.694)	60.4	66.5	0.269	829.96
PNI	0.606	(0.571, 0.641)	28.1	86.5	0.146	64.08
SIRI	0.656	(0.622, 0.691)	54.6	70.8	0.254	1.88
CAR	0.698	(0.665, 0.731)	59.3	72.7	0.320	0.32
mGPS	0.657	(0.622, 0.691)	64.3	65.7	–	–

### Univariate survival analysis of inflammatory markers and prognosis in NSCLC patients

**Figure 3:**
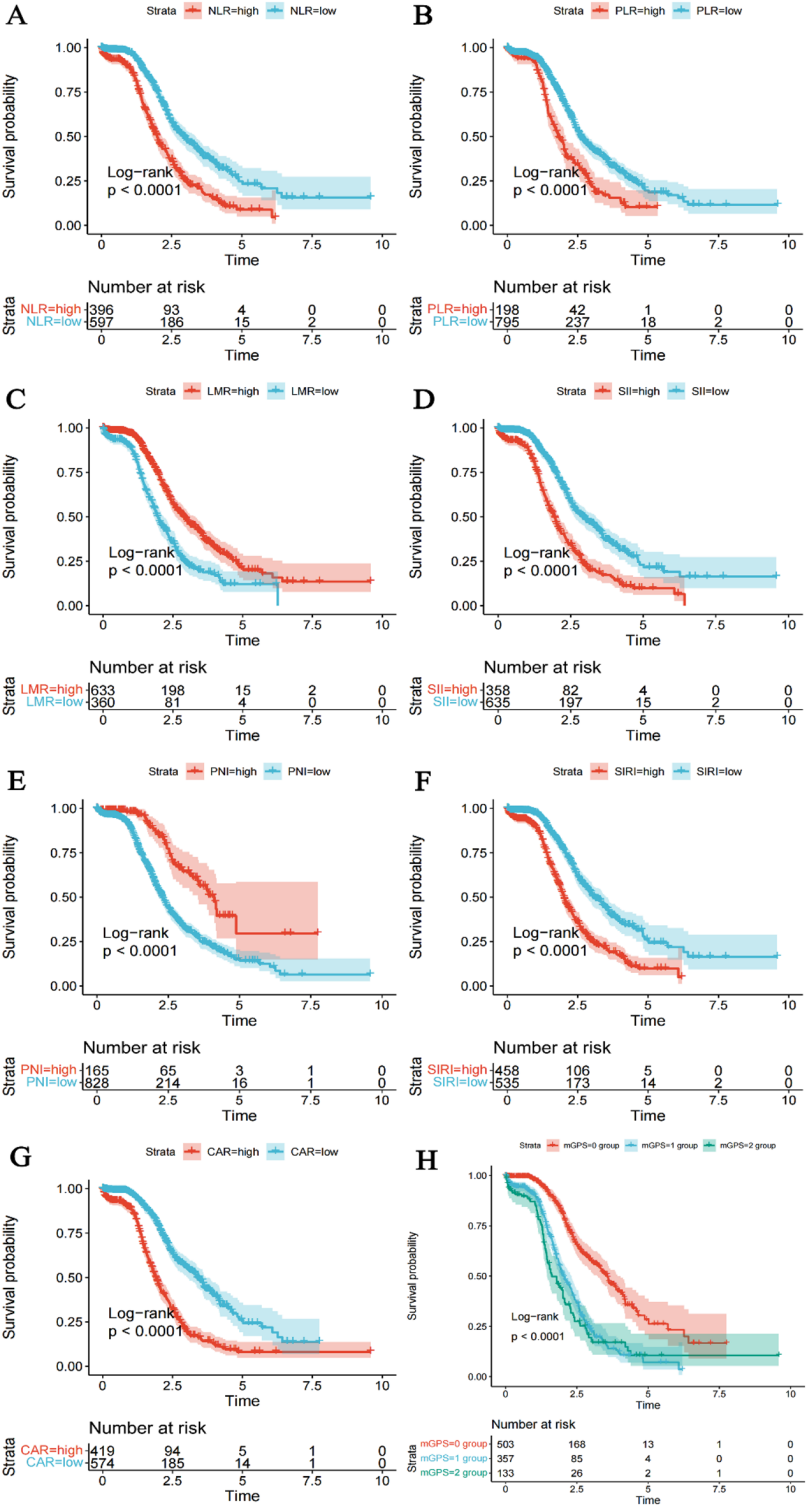
Kaplan–Meier survival curves of NSCLC patients with different subgroups of inflammatory markers. (**A**) NLR group (**B**) PLR group (**C**) LMR group (**D**) SII group (**E**) PNI group (**F**) SIRI group (**G**) CAR group (**H**) mGPS group.

Univariate Cox proportional hazard regression analyse showed that the eight inflammatory markers were independent factors influencing the prognosis of NSCLC patients when used as continuous variables, categorical variables (high versus low), and quartiles. NLR, PLR, SII, SIRI, and CAR showed an increasing risk of poor prognosis in groups Q2, Q3, and Q4 compared to group Q1. On the other hand, PNI and LMR demonstrated an increasing risk of poor prognosis in groups Q3, Q2, and Q1 compared to group Q4. Kaplan–Meier survival curves and Log-rank tests demonstrated that the high NLR group, high PLR group, low LMR group, high SII group, low PNI group, high SIRI group, high CAR group, mGPS 1 group, and mGPS 2 group were significantly associated with a poorer prognosis for NSCLC patients (*P* < 0.001). See Table 5 and Fig. 3.

**Table 5:** Relationship between inflammatory markers and OS in NSCLC patients.

Characteristic	Levels	Univariable	
		*HR* (95% CI)	*P*-value
NLR	Continuous	1.089 (1.067, 1.112)	**< 0.001**
	Cutoff value		
	≤ 3.42	Ref	Ref
	> 3.42	1.932 (1.607, 2.324)	**< 0.001**
	Quartiles		
	Q1 (< 2.08)	Ref	**< 0.001**
	Q2 (2.08–3.07)	1.600 (1.185, 2.161)	**000.2**
	Q3 (3.07–4.64)	2.080 (1.561, 2.770)	**< 0.001**
	Q4 (≥ 4.64)	2.894 (2.188, 3.828)	**< 0.001**
PLR	Continuous	1.002 (1.001, 1.002)	**< 0.001**
	Cutoff value		
	≤ 179.16	Ref	Ref
	> 179.16	1.526 (1.270, 1.834)	**< 0.001**
	Quartiles		
	Q1 (< 127.37)	Ref	**< 0.001**
	Q2 (127.37–172.32)	1.254 (0.946, 1.662)	0.116
	Q3 (172.32–232.71)	1.404 (1.069, 1.844)	**0.015**
	Q4 (≥ 232.71)	1.836 (1.412, 2.388)	**< 0.001**
LMR	Continuous	0.920 (0.873, 0.969)	**0.002**
	Cutoff value		
	≤ 3.85	2.043 (1.627, 2.565)	**< 0.001**
	> 3.85	Ref	Ref
	Quartiles		
	Q1 (< 1.98)	2.664 (2.017, 3.517)	**< 0.001**
	Q2 (1.98–3.03)	1.889 (1.419, 2.514)	**< 0.001**
	Q3 (3.03–4.18)	1.480 (1.104, 1.983)	**0.009**
	Q4 (≥ 4.18)	Ref	**< 0.001**
SII	Continuous	1.000 (1.000, 1.000)	**< 0.001**
	Cutoff value		
	≤ 829.96	Ref	Ref
	> 829.96	1.962 (1.627, 2.366)	**< 0.001**
	Quartiles		
	Q1 (< 489.04)	Ref	**< 0.001**
	Q2 (489.04–788.80)	1.503 (1.106, 2.044)	**0.009**
	Q3 (788.80–1314.71)	1.975 (1.484, 2.629)	**< 0.001**
	Q4 (≥ 1314.71)	2.740 (2.070, 3.627)	**< 0.001**
PNI	Continuous	0.970 (0.962, 0.979)	**< 0.001**
	Cutoff value		
	≤ 64.08	2.178 (1.666, 2.847)	**< 0.001**
	> 64.08	Ref	Ref
	Quartiles		
	Q1 (< 46.10)	2.407 (1.837, 3.155)	**< 0.001**
	Q2 (46.10–52.45)	1.569 (1.177, 2.092)	**0.002**
	Q3 (52.45–62.50)	1.636 (1.237, 2.166)	**0.001**
	Q4 (≥ 62.50)	Ref	**< 0.001**
SIRI	Continuous	1.108 (1.084, 1.133)	**< 0.001**
	Cutoff value		
	≤ 1.88	Ref	Ref
	> 1.88	2.050 (1.704, 2.465)	**< 0.001**
	Quartiles		
	Q1 (< 0.88)	Ref	**< 0.001**
	Q2 (0.88–1.54)	1.485 (1.097, 2.010)	**0.010**
	Q3 (1.54–2.86)	2.159 (1.614, 2.889)	**< 0.001**
	Q4 (≥ 2.86)	2.851 (2.153, 3.775)	**< 0.001**
CAR	Continuous	1.197 (1.148, 1.249)	**< 0.001**
	Cutoff value		
	≤ 0.32	Ref	Ref
	> 0.32	2.561 (2.123, 3.089)	**< 0.001**
	Quartiles		
	Q1 (< 0.06)	Ref	**< 0.001**
	Q2 (0.06–0.20)	1.802 (1.298, 2.502)	**< 0.001**
	Q3 (0.20–0.94)	3.109 (2.273, 4.251)	**< 0.001**
	Q4 (≥ 0.94)	4.106 (3.021, 5.582)	**< 0.001**
mGPS	0 score	Ref	**< 0.001**
	1 score	2.518 (2.050, 3.093)	**< 0.001**
	2 score	3.009 (2.307, 3.924)	**< 0.001**

Significant values are in bold.

### Analysing factors influencing the prognosis of NSCLC patients

Multivariate Cox proportional hazards model showed that age, tumor stage, smoking history, BMI, NLR, and CAR were independent prognostic factors. Specifically, age > 45 years, tumor stage III (*HR* = 3.301, 95% CI 1.909–5.710, *P* < 0.001), tumor stage IV (*HR* = 4.650, 95% CI 2.732–7.913, *P* < 0.001), smoking history (*HR* = 1.489, 95% CI 1.219–1.820, *P* < 0.001), low BMI level (*HR* = 0.971, 95% CI 0.944–0.999, *P* = 0.042), high NLR group (*HR* = 1.238, 95% CI 1.015–1.510, *P* = 0.035), high CAR group (*HR* = 1.729, 95% CI 1.408–2.124, *P* < 0.001) were associated with shorter OS in NSCLC patients. See Table 6 for details.

**Table 6:** Multivariate Cox regression analysis affecting the OS of NSCLC patients.

Characteristic	Levels	*B*	*SE*	*Wald χ*^2^	*P*-value	*HR* (95% CI)
Age	≤ 45	Ref	Ref	13.287	**0.001**	Ref
	46 –64	0.422	0.246	2.943	0.721	1.092 (0.673, 1.774)
	≥ 65	0.088	0.248	0.128	0.086	1.525 (0.942, 2.470)
Stage	I	Ref	Ref	44.525	**< 0.001**	Ref
	II	0.666	0.356	3.495	0.062	1.947 (0.968, 3.915)
	III	1.194	0.28	18.25	**< 0.001**	3.301 (1.909, 5.710)
	IV	1.537	0.271	32.085	**< 0.001**	4.650 (2.732, 7.913)
Smoking history	No	Ref	Ref	Ref	Ref	Ref
	Yes	0.398	0.102	15.139	**< 0.001**	1.489 (1.219, 1.820)
BMI	(median (IQR))	− 0.029	0.014	4.126	**0.042**	0.971 (0.944, 0.999)
NLR	≤ 3.42	Ref	Ref	Ref	Ref	Ref
	> 3.42	0.214	0.101	4.442	**0.035**	1.238 (1.015, 1.510)
CAR	≤ 0.32	Ref	Ref	Ref	Ref	Ref
	> 0.32	0.548	0.105	27.26	**< 0.001**	1.729 (1.408, 2.124)

Significant values are in bold.

### The relationship between NLR, CAR, and disease development in NSCLC patients

The *χ*^2^ and the Mann–Whitney U test were used to compare whether there was a statistical difference in each clinicopathological characteristic between the NLR and CAR subgroups of inflammatory markers. The results showed that compared to patients with a low NLR, those with a high NLR were predominantly aged ≥ 65 years, more likely to be male, had advanced tumor stage, a higher proportion had smoking history, a low BMI, and a higher proportion had an ECOG-PS of ≥ 2 (*P* < 0.05).Compared to patients with low CAR, those with high CAR were more often male, had advanced tumor stage, and had a higher proportion of individuals with a history of smoking and drinking, a low BMI, and a higher proportion had an ECOG-PS of ≥ 2 (*P* < 0.05).The high NLR and high CAR were significantly associated with an elevated inflammatory status (high neutrophil count, high monocyte count, low lymphocyte count, high platelet count, and high CRP) as well as malnutrition (low BMI, low KPS score, high ECOG-PS score and low ALB). See Table 7 for details.

**Table 7:** Relationship between NLR, CAR, and clinicopathological features of NSCLC patients.

Characteristic	Levels	NLR		
		High *n* = 414	Low *n* = 559	*P*-value
Age	≤ 45	26 (6.3)	31 (5.5)	**0.049**
	46 ~ 64	176 (42.5)	282 (50.4)	
	≥ 65	212 (51.2)	246 (44.0)	
Gender	Female	116 (28.0)	232 (41.5)	**< 0.001**
	Male	298 (72.0)	327 (58.5)	
Stage	I	18 (4.3)	122 (21.8)	**< 0.001**
	II	14 (3.4)	42 (7.5)	
	III	76 (18.4)	142 (25.4)	
	IV	306 (73.9)	253 (45.3)	
Primary site	Hilum	32 (7.7)	34 (6.1)	0.604
	Bronchial	16 (3.9)	22 (3.9)	
	Upper lobe of the lungs	195 (47.1)	290 (51.9)	
	Middle lobe of the lungs	29 (7.0)	34 (6.1)	
	Lower lobe of the lungs	142 (34.3)	179 (32.0)	
Pathology	Large cell carcinoma	3 (0.7)	2 (0.4)	**0.018**
	Adenocarcinoma	270 (65.2)	411 (73.5)	
	Squamous carcinoma	141 (34.1)	146 (26.1)	
Education	Elementary and below	140 (33.8)	165 (29.5)	0.495
	Middle School	128 (30.9)	175 (31.3)	
	High School	68 (16.4)	101 (18.1)	
	College and above	78 (18.8)	118 (21.1)	
Occupation	Self-employed	19 (4.6)	19 (3.4)	0.071
	Workers	50 (12.1)	69 (12.3)	
	Farmers and herdsmen	89 (21.5)	89 (15.9)	
	Others	77 (18.6)	122 (21.8)	
	Institutionalized	26 (6.3)	41 (7.3)	
	Retired	115 (27.8)	139 (24.9)	
	Unemployed	15 (3.6)	38 (6.8)	
	Employee	23 (5.6)	42 (7.5)	
Smoking history	No	156 (37.7)	285 (51.0)	**< 0.001**
	Yes	258 (62.3)	274 (49.0)	
Drinking history	No	272 (65.7)	390 (69.8)	0.179
	Yes	142 (34.3)	169 (30.2)	
BMI	(median (IQR))	23.18 (20.98, 25.19)	24.22 (22.04, 26.45)	**< 0.001**
BSA	(median (IQR))	1.70 (1.59, 1.83)	1.71 (1.60, 1.84)	0.383
KPS score	(median (IQR))	90 (80, 90)	90 (90, 90)	**< 0.001**
ECOG-PS	0–1	380 (91.79)	549 (98.21)	**< 0.001**
	≥ 2	34 (8.21)	10 (1.79)	
Neutrophil	(median (IQR))	6.37 (5.02, 8.70)	3.82 (3.02, 4.77)	**< 0.001**
Lymphocyte	(median (IQR))	1.18 (0.92, 1.49)	1.74 (1.43, 2.16)	**< 0.001**
Monocyte	(median (IQR))	0.62 (0.48, 0.80)	0.46 (0.35, 0.60)	**< 0.001**
Platelet	(median (IQR))	269.00 (214.50, 360.00)	249.00 (201.00, 295.00)	**< 0.001**
CRP	(median (IQR))	25.00 (7.00, 75.00)	5.00 (1.84, 16.00)	**< 0.001**
ALB	(median (IQR))	41.40 (35.58, 48.63)	46.10 (40.60, 61.50)	**< 0.001**

Characteristic	Levels	CAR		
		High *n* = 413	Low *n* = 560	*P*-value
Age	≤ 45	20 (4.8)	37 (6.6)	0.458
	46 ~ 64	193 (46.7)	265 (47.3)	
	≥ 65	200 (48.4)	258 (46.1)	
Gender	Female	90 (21.8)	258 (46.1)	**< 0.001**
	Male	323 (78.2)	302 (53.9)	
Stage	I	18 (4.4)	122 (21.8)	**< 0.001**
	II	11 (2.7)	45 (8.0)	
	III	85 (20.6)	133 (23.8)	
	IV	299 (72.4)	260 (46.4)	
Primary site	Hilum	39 (9.4)	27 (4.8)	**0.011**
	Bronchial	22 (5.3)	16 (2.9)	
	Upper lobe of the lungs	201 (48.7)	284 (50.7)	
	Middle lobe of the lungs	26 (6.3)	37 (6.6)	
	Lower lobe of the lungs	125 (30.3)	196 (35.0)	
Pathology	Large cell carcinoma	2 (0.5)	3 (0.5)	**< 0.001**
	Adenocarcinoma	250 (60.5)	431 (77.0)	
	Squamous Carcinoma	161 (39.0)	126 (22.5)	
Education	Elementary and below	139 (33.7)	166 (29.6)	0.580
	Middle School	127 (30.8)	176 (31.4)	
	High School	68 (16.5)	101 (18.0)	
	College and above	79 (19.1)	117 (20.9)	
Occupation	Self-employed	20 (4.8)	18 (3.2)	**0.007**
	Workers	54 (13.1)	65 (11.6)	
	Farmers and herdsmen	90 (21.8)	88 (15.7)	
	Others	86 (20.8)	113 (20.2)	
	Institutionalized	18 (4.4)	49 (8.8)	
	Retired	106 (25.7)	148 (26.4)	
	Unemployed	14 (3.4)	39 (7.0)	
	Employee	25 (6.1)	40 (7.1)	
Smoking history	No	124 (30.0)	317 (56.6)	**< 0.001**
	Yes	289 (70.0)	243 (43.4)	
Drinking history	No	245 (59.3)	417 (74.5)	**< 0.001**
	Yes	168 (40.7)	143 (25.5)	
BMI	(median (IQR))	23.19 (21.26, 25.45)	24.10 (21.94, 26.30)	**0.001**
BSA	(median (IQR))	1.72 (1.61, 1.84)	1.70 (1.58, 1.83)	0.143
KPS score	(median (IQR))	90 (80, 90)	90 (90, 90)	**< 0.001**
ECOG-PS	0–1	380 (92.01)	549 (98.04)	**< 0.001**
	≥ 2	33 (7.99)	11 (1.96)	
Neutrophil	(median (IQR))	5.78 (4.52, 8.09)	4.02 (3.14, 5.19)	**< 0.001**
Lymphocyte	(median (IQR))	1.39 (1.05, 1.83)	1.59 (1.23, 1.96)	**< 0.001**
Monocyte	(median (IQR))	0.64 (0.49, 0.82)	0.45 (0.35, 0.58)	**< 0.001**
Platelet	(median (IQR))	287.00 (233.50, 374.50)	242.00 (195.25, 284.00)	**< 0.001**
CRP	(median (IQR))	48.08 (25.00, 95.53)	3.65 (1.35, 6.60)	**< 0.001**
ALB	(median (IQR))	40.00 (33.95, 46.45)	47.39 (41.70, 57.90)	**< 0.001**

Significant values are in bold.

### Constructing nomogram and calibration curves

**Figure 4:**
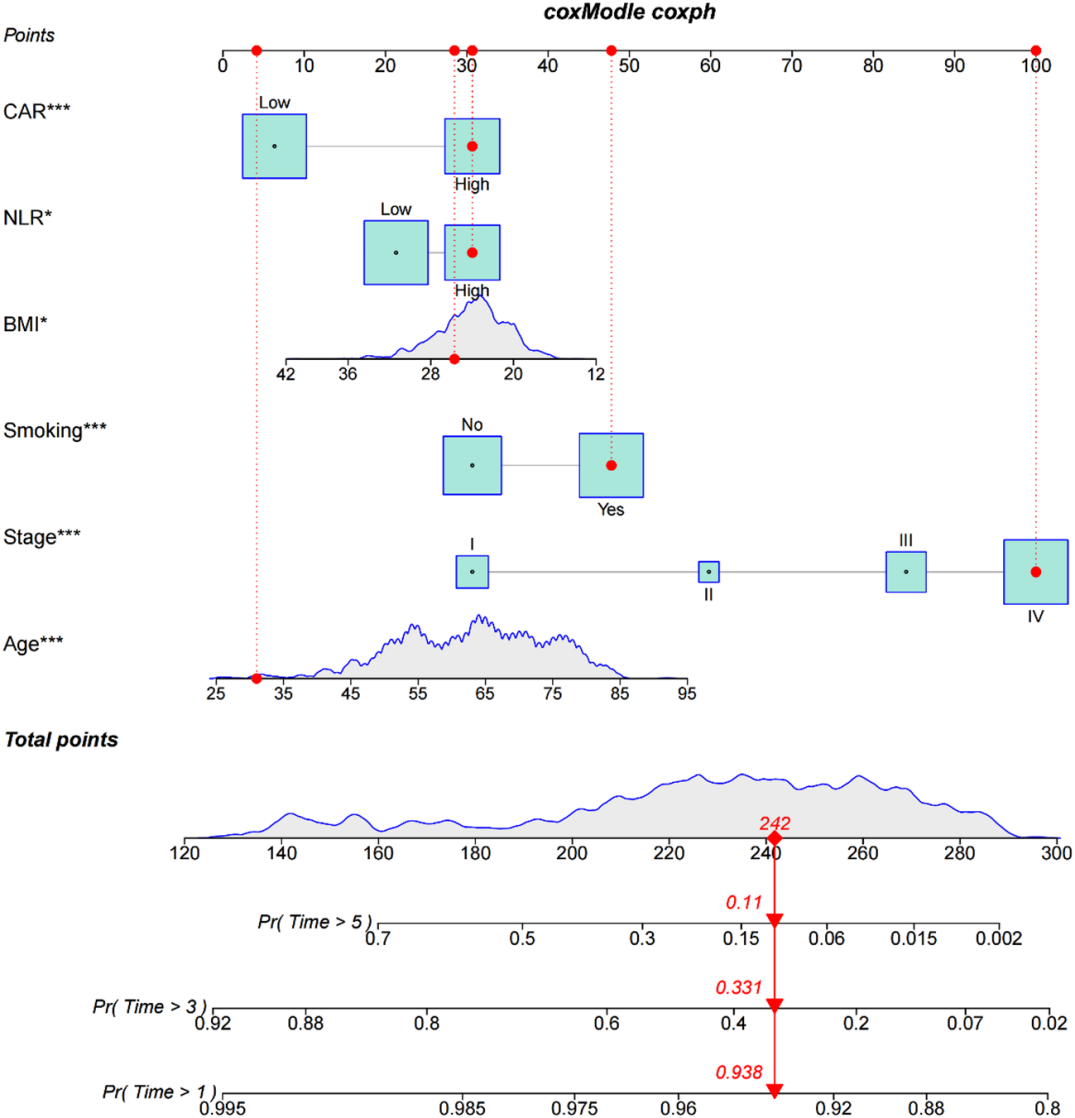
Nomogram predicting OS in NSCLC patients.

**Figure 5:**
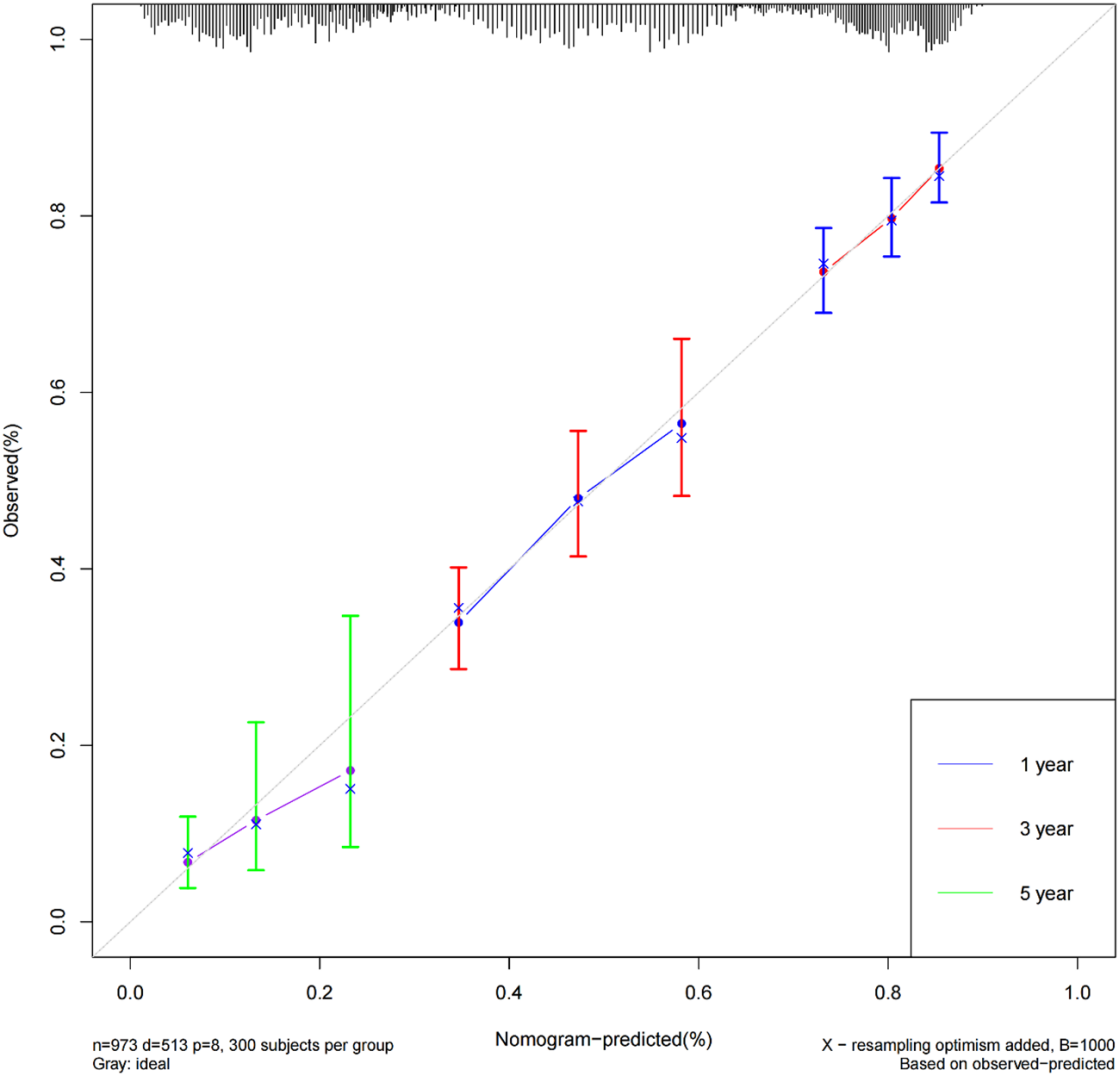
Calibration curves for 1-, 3-, and 5-year survival nomogram in NSCLC patients.

Integrating the data on six characteristics, including age, tumor stage, smoking history, BMI, NLR, CAR, and OS and survival status of 973 NSCLC patients, the Cox method was used to construct a nomogram model (Fig. 4). The overall C-index of the model was found to be 0.708 (95% *CI* 0.680–0.731), indicating that the inflammatory markers used in the construction of the nomogram model have a good predictive ability. The Bootstrap method was used to internally validate the nomogram by plotting the calibration curves for 1-year, 3-year, and 5-year. The results shows that the predicted curves for 1-year and 3-year fit well with the ideal curve, while the curve for 5-year has the worst overlap. Overall, the nomogram demonstrated good predictive stability and consistency (Fig. 5).

## Discussion

Inflammation plays a crucial role in the development and progression of tumors. It promotes the proliferation and migration of tumor cells, stimulates angiogenesis, and suppresses the body's natural anti-tumor immune response^9^. The tumor-related inflammatory status not only affects its local microenvironment but also promotes or maintains the release of various inflammatory components into the bloodstream at the systemic level. This, in turn, triggers a cancer-induced systemic inflammatory response^10^. Accumulating evidence proves that systemic inflammatory responses are immune responses that promote cancer. Hematologic products of inflammatory processes can be considered potential biomarkers^11^. In recent years, systemic inflammatory markers consisting of peripheral blood markers such as NLR, PLR, and mGPS, have been utilized not only for predicting the prognosis of different malignant tumors, but also for forecasting disease recurrence and treatment response^12–14^. Inflammatory markers have become a new research focus due to their significant role in guiding clinical practice, as well as their simple and convenient measurement methods and affordability.

This study systematically and comprehensively compared the predictive value of eight systemic inflammatory markers (NLR, PLR, LMR, SII, PNI, SIRI, CAR, mGPS) composed of peripheral blood markers for the prognosis of NSCLC patients. Univariate Cox regression analysis revealed that the eight systemic inflammatory markers were independently associated with the prognosis of NSCLC patients. Further use of multivariate Cox regression analysis revealed that age, tumor stage, smoking history, BMI, NLR, and CAR independently predicted OS in NSCLC patients. Based on these six metrics, nomogram was constructed and validated to predict the prognosis for 1-, 3-, and 5-year survival of NSCLC patients. Nomogram models can integrate the actual clinical characteristics of patients and additional prognostic factors, and they have been widely used for tumor prognosis^15^. Compared to traditional predictive models, simple and straightforward nomogram prediction models are easier to apply in clinical decision-making and can predict individual clinical outcomes based on individual characteristics, thus facilitating personalised treatment^16^. The results showed that the C-index of the nomogram for predicting OS of NSCLC patients was 0.708, indicating that the model predicted well.

The ECOG-PS score is widely utilized to quantify the performance status of cancer patients^17^. Higher ECOG-PS scores indicate poorer performance status and have been demonstrated to be prognostic and predictive for cancer patients^18^. The NLR is calculated by dividing the neutrophil count by the lymphocyte count in peripheral blood. In this study, we found that higher NLR values were associated with a worse prognosis in NSCLC patients. Additionally, high NLR was significantly associated with poor outcomes such as malnutrition, a high inflammatory state, advanced tumor stage, and high ECOG-PS scores. This supports the theory that poor performance status is commonly observed in advanced cancer clinics and validates the ability of NLR to serve as an inflammatory marker indicative of cancer severity. Shi et al.^19^ reported on the correlation between NLR as an index of immunoinflammation and the prognosis of NSCLC patients. Neutrophils are central mediators of the innate immune response and have emerged as key players in many inflammatory and immune-mediated diseases, including cancer, due to their pro-inflammatory effects^20^. In general, neutrophils play a dual role in lung cancer. They act as inhibitors in the early stages of cancer development but promote cancer growth and metastasis in the middle and late stages. Neutrophils are significantly associated with a higher rate of recurrence and a poorer prognosis for survival in lung cancer^21^. Neutrophils can contribute to tumor development through direct mechanisms, such as promoting genetic instability and cell proliferation, or through indirect mechanisms, such as promoting metastatic spread or suppressing anti-tumor immune responses^22^. Lymphocytes play a crucial role in the body's immune system. Tumor-infiltrating lymphocytes have the ability to induce cytotoxic cell death and inhibit the proliferation and migration of tumor cells by regulating immune interactions^23^. According to this mechanism, an increased lymphocyte count inhibits tumorigenesis and progression and is associated with a good prognosis for cancer^24^. Conversely, a low lymphocyte count indicates a weakened anti-tumor immune response, which may indicate a poor prognosis^25^. High NLR values indicate that the patient has neutrophilia or lymphocytopenia, suggesting that the patient is in a high inflammatory status, which can lead to a worse prognosis.

The CAR is calculated based on the levels of CRP and ALB in peripheral blood. It combines the body's inflammatory response and nutritional status. It serves as an inflammation marker related to disease progression and nutritional status. In this study, we found that higher CAR values were associated with a worse prognosis in NSCLC patients. Additionally, high CRP was also significantly associated with poor outcomes, including malnutrition, a high inflammatory state, advanced tumor stage, and poorer performance status. CRP is a classical acute-phase response protein, the production of which is closely linked to inflammatory stimulation of hepatocytes and macrophages^26^. It is rapidly elevated during acute inflammatory responses. CRP has been shown to be elevated in many cancers (e.g. NSCLC) and is correlated with poor outcomes. This increase in CRP has been attributed to factors such as tumor necrosis and tumor inflammation^27^. Serum Alb is primarily synthesized and secreted by the liver. It plays a crucial role in maintaining osmotic pressure and providing nutrition. Additionally, it not only reflects the body's nutritional status but also exhibits anti-inflammatory effects. Moreover, it can enhance the body's immune response, thereby potentially playing a role in anti-tumor activity. Consequently, assessing the prognosis of NSCLC patients is valuable^28^. A cohort study showed that patients with high Alb levels in lung cancer had a better prognosis^29^. A high CAR value indicates that the patient has an increased CRP or decreased Alb, suggesting a chronic inflammatory response or malnutrition. This can weaken the body's defense system and diminish the effect of systemic biopharmaceuticals, leading to a poor prognosis.

In addition to inflammatory markers, age, tumor stage, smoking history, and BMI were also identified as risk factors for survival prognosis in NSCLC patients, which is consistent with previous research findings. From 1990 to 2019, the incidence and mortality rates of lung cancer among urban and rural residents in China increased with age, imposing significant health and economic burdens on elderly lung cancer patients and society^30^. Tumor stage remains the most reliable prognostic factor for OS. The higher the stage, the lower the survival rate for patients. In stage IV primary lung cancer, metastatic sites can include the bone, brain, liver, and intraparenchymal sites. These metastases have a significant impact on survival and prognosis^31^. Smoking is a well-known risk factor for lung cancer worldwide. It has been found that over 80% of NSCLC patients have a history of smoking. Furthermore, continuing to smoke after being diagnosed with cancer can lead to an increase in post-treatment complications and recurrences, as well as a decrease in post-treatment survival^32^. It is now well-documented that BMI is associated with a reduced risk of lung cancer, and a higher BMI reduces postoperative morbidity and/or mortality in patients^33^. The clinicopathological features mentioned above, along with inflammatory markers, may offer a more precise and personalized prognostic assessment for NSCLC patients.

The inflammatory markers NLR and CAR, derived from the assessment in this study, effectively reflect the inflammatory and nutritional status of NSCLC patients. This suggests that patients with elevated levels of inflammation and physical malnutrition are more likely to experience unfavorable outcomes and should be given clinical attention. Meanwhile, routine blood tests are characterized by their low cost and easy accessibility. The hematological products of the inflammatory process can be used as potential biomarkers to determine the poor prognosis of NSCLC in a timely manner. This has a broad clinical application prospect. This study also has limitations. First, this study was a retrospective analysis with data from a single cancer center. The possibility of residual and unmeasured confounding cannot be completely excluded. The results of the study need to be confirmed by multicenter, large-sample, long-term follow-up clinical studies to validate and assess the prognostic ability of these inflammatory markers. Secondly, measurement bias exists due to the fact that peripheral blood cell counts were conducted only once. In addition, inflammatory markers may be influenced by inflammation, medications, complications, and other factors. Finally, because the data collection was not comprehensive enough to gather information on various treatment modalities for NSCLC patients, as well as details on diagnosis, treatment follow-up, and the relationship between inflammatory markers and metastasis, recurrence, and prognosis of NSCLC patients before and after different treatments, further analysis could not be conducted.

## Conclusions

In summary, hematological indicators are easily obtained in the clinic due to their simple operation and low cost. In addition, utilizing inflammatory markers obtained from peripheral blood tests as prognostic markers may assist in alleviating the financial burden on patients and social healthcare resources. NLR and CAR, identified in this study, are independent factors that influence the OS of NSCLC patients. CAR is the most reliable indicator of systemic inflammation for predicting the prognosis of NSCLC. Additionally, the nomogram model, which incorporates age, pathological stage, smoking history, and BMI, can effectively predict the prognosis of NSCLC patients and provide guidance for clinical treatment and follow-up monitoring.

### Supplementary Information

Supplementary Information.

## Data Availability

All data generated or analyzed during this study are included in this article and its supplementary material files. Further enquiries can be directed to the corresponding author.
